# Threatening illness perception and associated factors in early-stage relapsing-remitting multiple sclerosis

**DOI:** 10.3389/fpsyt.2025.1565150

**Published:** 2025-07-08

**Authors:** Rocío Gómez-Ballesteros, Susana Sainz de la Maza, Mónica Borges, Jesús Martín-Martínez, Javier Sotoca, Ana Alonso, Ana B. Caminero, Laura Borrega, José L. Sánchez-Menoyo, Francisco J. Barrero-Hernández, Carmen Calles, Luis Brieva, María R. Blasco-Quílez, Julio Dotor García-Soto, Ana Rodríguez-Regal, Laura Navarro-Cantó, Eduardo Agüera, Moisés Garcés-Redondo, Olga Carmona, Laura Gabaldón-Torres, Lucía Forero, Mariona Hervás, Tamara Castillo-Triviño, Jorge Maurino

**Affiliations:** ^1^ Medical Department, Roche Farma, Madrid, Spain; ^2^ Department of Neurology, Hospital Universitario Ramón y Cajal, Madrid, Spain; ^3^ Department of Neurology, Hospital Universitario Virgen Macarena, Sevilla, Spain; ^4^ Department of Neurology, Hospital Universitario Miguel Servet, Zaragoza, Spain; ^5^ Department of Neurology, Hospital Universitari Mútua Terrassa, Terrassa, Spain; ^6^ Department of Neurology, Hospital Regional Universitario de Málaga, Málaga, Spain; ^7^ Department of Neurology, Complejo Asistencial de Ávila, Ávila, Spain; ^8^ Department of Neurology, Hospital Universitario Fundación Alcorcón, Alcorcón, Spain; ^9^ Department of Neurology, Hospital de Galdakao-Usansolo, Galdakao, Spain; ^10^ Department of Neurology, Hospital Universitario Clínico San Cecilio, Granada, Spain; ^11^ Department of Neurology, Hospital Universitari Son Espases, Palma de Mallorca, Spain; ^12^ Department of Neurology, Hospital Arnau de Vilanova de Lleida, UdL Medicine Department, Instituto de Investigación Biomédica de Lleida (IRBLleida), Lleida, Spain; ^13^ Department of Neurology, Hospital Universitario Puerta de Hierro, Madrid, Spain; ^14^ Department of Neurology, Complexo Hospitalario Universitario de Pontevedra, Pontevedra, Spain; ^15^ Department of Neurology, Hospital General Universitario de Elche, Elche, Spain; ^16^ Department of Neurology, Hospital Universitario Reina Sofía, Córdoba, Spain; ^17^ Department of Neurology, Hospital Clínico Universitario Lozano Blesa, Zaragoza, Spain; ^18^ Department of Neurology, Fundació Salut Empordà, Figueres, Spain; ^19^ Department of Neurology, Hospital Francesc de Borja, Gandía, Spain; ^20^ Department of Neurology, Hospital Universitario Puerta del Mar, Cádiz, Spain; ^21^ Department of Neurology, Consorci Corporació Sanitària Parc Taulí, Sabadell, Spain; ^22^ Department of Neurology, Hospital Universitario Donostia, San Sebastián, Spain

**Keywords:** relapsing-remitting multiple sclerosis, illness perception, early-stage, patient-reported outcome measures, individualized interventions, psychological factors

## Abstract

**Background:**

Multiple sclerosis is one of the most common causes of neurological disability in young adults, with major consequences for their future lives. Patients with early-stage relapsing-remitting multiple sclerosis (RRMS) experience uncertainty and intense emotions as the diagnosis is disclosed. Illness perception at this point can influence levels of adjustment, coping strategies, treatment adherence, and well-being of the patient. However, there is limited information on patient illness perception surrounding the diagnosis.

**Objective:**

The aim of this study was to assess illness perception and associated factors in early-stage RRMS patients.

**Methods:**

A multicenter, non-interventional study was conducted. Adult patients with a diagnosis of RRMS, a disease duration of ≤ 3 years, and an Expanded Disability Status Scale (EDSS) score of 0-5.5 were included. The Brief-Illness Perception Questionnaire (B-IPQ) was used to assess the patients’ cognitive and emotional representations of their illness. Different patient-reported measures were used to gather information on pain, fatigue, mood/anxiety, quality of life, symptom severity, feelings of hopelessness, perception of stigma, cognition, hand dexterity, gait, and workplace difficulties. A multivariate logistic regression analysis was performed to assess the association between the patients’ illness perception and demographic and clinical characteristics, as well as patient-reported outcomes.

**Results:**

A total of 189 patients were included (mean age: 36.1 ± 9.4 years, 71.4% females, mean disease duration: 1.4 ± 0.8 years). The median EDSS score was 1.0 (interquartile range: 0.0-2.0). A total of 36.5% of the patients (n=69/189) had a moderate-to-high threatening illness perception, and 45.5% thought that their disease was caused by psychological factors. Higher EDSS scores, symptom severity, poorer psychological quality of life, perception of stigma, and greater hopelessness were predictors of moderate-to-high threatening illness perception.

**Conclusions:**

Threatening illness perceptions are common among patients with early-stage RRMS. Identifying these beliefs and their associated factors, and establishing individualized interventions, may help patients deal with their condition.

## 1 Introduction

Multiple sclerosis (MS) is a chronic demyelinating autoimmune disease that constitutes one of the most common causes of neurological disability in young adults (1). There are approximately two million people worldwide affected by MS, with a prevalence of 23.9 cases per 100,000 population and 62,000 individuals diagnosed with MS every year (2). It manifests through different symptoms such as visual impairment, gait problems, sensory disturbance, fatigue and cognitive problems, among others (3–5). Most patients have a relapsing-remitting form of multiple sclerosis (RRMS), while the rest have primary progressive or secondary progressive forms of MS. Patients with RRMS face the diagnosis in early-to-mid adulthood, with profound consequences in their lives through the disruption of goals, employment, relationships, family planning or social activities (6, 7). The disease can also lead to social cognition deficits due cognitive decline and emotional impairment in patients, deriving in difficulties interpreting social cues, emotions, and establishing meaningful relationships, with an impact in patients’ quality of life (8). The unknown etiology of the disease, combined with the unpredictability of relapses, variable clinical course, and chronic progression without a cure, induces a sense of uncertainty in patients, potentially affecting their perception of the disease and overall well-being (9–12).

Intense emotions, anxiety and depression are also common in the period surrounding the diagnosis, and can affect patient understanding and adjustment to the disease (13, 14). In the event of a health problem or following diagnosis, patients develop their own beliefs and perceptions about the illness, related symptomatology, timeline course, causes, consequences, control through personal actions and treatments, individual emotional response, and coherence (15, 16). Illness perception at this point can influence the levels of adaptation, coping strategies, treatment adherence, and well-being of the patients (16–18). Understanding the illness perceptions of the patients and addressing them at the start of the disease may help them deal with their condition and adjustment to the disease over the long-term, pursuing better quality of life and well-being. However, there is scarce evidence on illness perceptions in early-stage RRMS patients with low levels of disability. Thus, the aim of the present study was to describe patient illness perceptions and assess the factors associated with them in a population recently diagnosed of RRMS.

## 2 Methods

### 2.1 Study design

A multicenter, non-interventional, cross-sectional study (MS-ONSET study) was carried out. Inclusion criteria included an age of 18 years or older, a diagnosis of RRMS according to the 2017 revised McDonald criteria, a disease duration of ≤3 years, and an Expanded Disability Status Scale (EDSS) score of 0 to 5.5 (19, 20). Patients not able to understand or complete the study questionnaires according to neurologist criteria, those who had a relapse recently and those who were not stable on their treatment were excluded from the sample. Patients were consecutively recruited in the context of their follow-up visits at 21 hospital-based Neuroimmunology clinics. When attending the follow-up visits, patients fulfilling inclusion criteria were offered to participate in the study by neurologists, and after signing the informed consent they were included in the study and completed all the questionnaires at the hospital. The EDSS was assessed by their treating neurologists.

### 2.2 Outcome measures

The Brief-Illness Perception Questionnaire (B-IPQ) was used to assess the patients’ cognitive and emotional representations of their illness (21, 22). It consists of 8 items graded on a linear 0–10 response scale. Each item of the B-IPQ assesses one dimension of illness perception, including the consequences, timeline or duration of disease, personal control, treatment control, identity or symptoms, concerns, coherence or understanding, and emotional impact. An overall score can be calculated by adding all items and reversing scores of items of personal control, treatment control, and coherence/understanding. Higher scores indicate a more threatening illness perception. A ninth item consisting of an open question addresses the patients’ thoughts about the cause of their illness (21, 22). Moderate-to-high experience of threat is defined as a B-IPQ score of at least 42 points (23).

Different patient-reported measures were used to gather information on pain [Visual Analogue Scale (VAS)] (24), fatigue [5-item Modified Fatigue Impact Scale (MFIS-5)] (25), mood and anxiety [Hospital Anxiety and Depression Scale (HADS)] (26), quality of life [Multiple Sclerosis Impact Scale (MSIS-29)] (27), symptom severity [SymptoMScreen (SyMS)] (28, 29), feelings of hopelessness [State-Trait Hopelessness Scale (STHS)] (30), perception of stigma [Stigma Scale for Chronic Illness 8-item version (SSCI-8)] (31, 32), gait perception [Multiple Sclerosis Walking Scale (MSWS-12)] (33), manual dexterity perception [NeuroQoL Upper Extremity (NeuroQoL-UE)] (34), cognition [Perceived Deficits Questionnaire (PDQ-5)] (35), and workplace difficulties [Multiple Sclerosis Working Difficulties Questionnaire (MSWDQ-23)] (36) (**Table 1**).

**Table 1:** Outcome measures.

Outcome	Measure	Scoring and interpretation	Range
Symptom severity	SyMS	The SyMS assesses symptom severity across 12 neurological domains. Each item is assessed on a 7-point Likert scale from 0 (not at all affected) to 6 (total limitation). Higher scores indicate more severe symptom involvement.	0-72
Disability	EDSS	The EDSS is a measure to quantify disability in 8 functional systems. It is an ordinal rating system ranging from 0 (normal) to 10 (death), in 0.5-increment intervals.	0-10
Fatigue	MFIS-5	The MFIS-5 assesses physical, cognitive, and psychosocial components of fatigue. Each item scores on a 5-point Likert scale from 0 (never) to 4 (almost always). Higher scores indicate more severe fatigue.	0-20
Pain	VAS	Visual analogue scale, with higher scores indicating a higher level of pain.	0-100
Mood and anxiety	HADS	The HADS is a 14-item, self-assessment scale to measure symptoms of anxiety and depression. Each item is scored on a 4-point Likert scale from 0 to 3. A total subscale score >10 indicates a probable case of anxiety or depression, respectively.	0-21
Quality of life	MSIS-29	The MSIS-29 measures the impact of multiple sclerosis on health-related quality of life. It consists of two composite domains including physical (20 items) and psychological impacts (9 items). Items are rated using a 4-point Likert scale from 1 (not at all) to 4 (extremely). Higher scores indicate greater impact.	20-80 (physical) 9-36 (psychological)
Hopelessness	STHS	The STHS is an instrument to differentiate trait (13 items) and state (10 items) hopelessness, where each subscale is measured on a 4-point Likert scale ranging from 1 (strongly disagree) to 4 (strongly agree). Higher scores indicate higher levels of hopelessness. A cut-off score ≥ 1.8 is used to define the presence of moderate-to-severe state hopelessness.	1-4
Stigma	SSCI-8	The SSCI-8 assesses internalized and experienced stigma across neurological conditions. Each item is rated on a 5-point Likert scale from 1 (never) to 5 (always). A cut-off score > 8 indicates the presence of stigmatization.	8-40
Workplace difficulties	MSWDQ-23	The MSWDQ-23 assesses the extent of physical, psychological/cognitive, and external difficulties experienced in the workplace. Each item is scored on a 5-point Likert scale from 0 (never) to 4 (almost always). All the subscales and the total scale are scored as a percentage by summing the observed item scores, divided by the total possible item scores in each subscale, then multiplying the value by 100. Higher scores indicate greater difficulties.	0-100
Hand dexterity	NeuroQoL-UE	The NeuroQoL-UE assesses patient ability to carry out activities involving digital (e.g., making a phone call), manual, and reach-related functions (e.g., washing and drying themselves). It is an 8-item form rated from 1 (I cannot do it) to 5 (I can do it without difficulty), with higher scores reflecting better upper extremity motor function.	8-40
Gait	MSWS-12	The MSWS-12 assesses the difficulties experienced by individuals in walking function and quality. Each of the 12 items are rated from 1 (not at all) to 5 (extremely).	12-60, transformed into 0-100
Cognition	PDQ-5	The PDQ-5 assesses cognitive complaints on four subscales. Each of the 5 items are scored from 0 (never) to 5 (very often). Higher scores indicate greater difficulties.	0-5

EDSS, Expanded Disability Status Scale; HADS, Hospital Anxiety and Depression Scale; MFIS-5, 5-item Modified Fatigue Impact Scale; MSIS-29, Multiple Sclerosis Impact Scale; MSWDQ-23, 23-item Multiple Sclerosis Working Difficulties Questionnaire; MSWS-12: Multiple Sclerosis Walking Scale; NeuroQoL-UE: NeuroQoL-Upper Extremity; PDQ-5, 5-item Perceived Deficit Questionnaire; SSCI-8, Stigma Scale for Chronic Illness; SyMS, SymptoMScreen; STHS, State-Trait Hopelessness Scale; SymptoMScreen; VAS, Visual Analogue Scale. All items are patient-reported measures except for EDSS.

### 2.3 Methodological approach

Demographic and clinical characteristics were reported as frequencies (percentages) and means (standard deviations). Bivariate analyses were performed using logistic regression to assess the association between categorized B-IPQ (dependent variable) and demographic parameters, clinical characteristics, and patient perspectives. Subsequently, a multivariate logistic regression analysis was performed taking each of the described variables as the dependent variable and each of the values found to be significant (<0.10) in the previous analysis as the independent variables. These variables were further selected through stepwise regression using the Akaike information criterion (AIC).

## 3 Results

A total of 189 patients were included in the study. The mean age was 36.1 years, and 71.4% were females. The mean disease duration was 1.4 years, and the median EDSS score was 1.0. Symptom severity was low, but the patients reported that their psychological quality of life was somewhat impacted, with 56.6% perceiving stigma and almost 25% of the patients being probable anxiety cases. The sociodemographic and clinical characteristics of the sample are shown in **Table 2**.

**Table 2:** Sociodemographic and clinical characteristics of the study population.

Variable	N=189
Age, years, mean (SD)	36.1 (9.4)
Sex (female), n (%)	135 (71.4)
Education, n (%)	
University	151 (79.9)
Living status, n (%)	
With partner/family members	164 (86.8)
Working status, n (%)	
Partial or full-time employed	130 (68.8)
Time since disease onset, years, median (IQR)	1.4 (0.7, 2.1)
Number of relapses since first attack, mean (SD)	1.8 (8.4)
EDSS score, median (IQR)	1.0 (0, 2.0)
SyMS score, mean (SD)	12.0 (10.8)
B-IPQ score, mean (SD)	38.0 (11.8)
Moderate-to-high threatening perception, n (%)	69 (36.5)
MSIS-29	
Physical impact score, mean (SD)	29.2 (11.3)
Psychological impact score, mean (SD)	17.2 (6.6)
MFIS-5 score, mean (SD)	6.2 (5.1)
Pain VAS score, mean (SD)	14.1 (23.1)
STHS	
Trait score, mean (SD)	2.0 (0.5)
State score, mean (SD)	2.0 (0.5)
State score ≥ 1.8, n (%)	124 (65.6)
HADS	
Anxiety score, mean (SD)	7.8 (4.3)
Depression score, mean (SD)	4.1 (3.9)
Anxiety, probable cases, n (%)	47 (24.9)
Depression, probable cases, n (%)	13 (6.9)
SSCI-8 score, mean (SD)	10.4 (3.9)
>8, n (%)	107 (56.6)
MSWS-12 global score, median (IQR)	6.3 (0-22.9)
NeuroQoL-UE global score, mean (SD)	38.5 (3.7)
PDQ-5 score, mean (SD)	4.9 (4.4)
MSWDQ-23 total score, median (IQR)	11.4 (4.6, 27.3)^a^
Physical barriers, median (IQR)	9.4 (3.1, 25.0)^a^
Psychological/cognitive barriers, median (IQR)	11.4 (4.5, 27.3)^a^
External barriers, median (IQR)	12.5 (0, 37.5)^a^

B-IPQ, Brief Illness Perception Questionnaire; EDSS, Expanded Disability Status Scale; HADS, Hospital Anxiety and Depression Scale; IQR, Interquartile range; MFIS-5, 5-item Modified Fatigue Scale; MSIS-29, Multiple Sclerosis Impact Scale; MSWDQ-23, 23-item Multiple Sclerosis Work Difficulties Questionnaire; MSWS-12, Multiple Sclerosis Walking Scale; NeuroQoL-UE, NeuroQoL Upper Extremity; PDQ-5, Perceived Deficits Questionnaire; SD, Standard deviation; SSCI-8, Stigma Scale for Chronic Illness; STHS, State-Trait Hopelessness Scale; SyMS, SymptoMScreen; VAS, Visual Analogue Scale. ^a^N=183.

**Figure 1:**
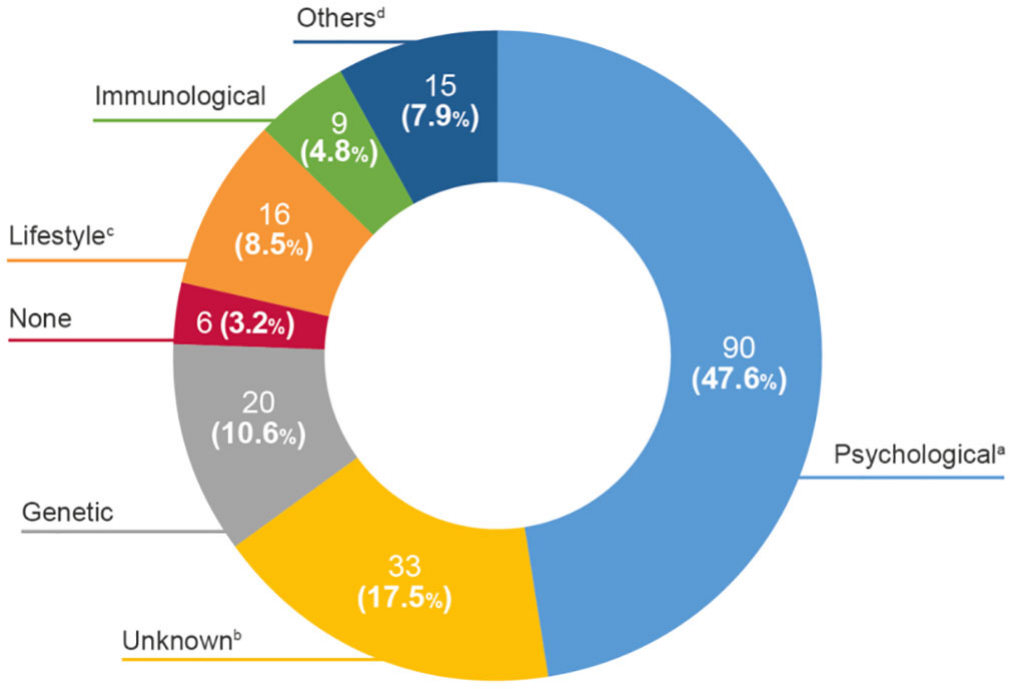
Causes of multiple sclerosis reported by patients. ^a^Psychological includes emotional causes, depression, anxiety, nervousness, stress; ^b^Unknown includes chance or bad luck; ^c^Lifestyle includes smoking, diet, lack of physical exercise; ^d^others includes severe sunstroke, car accident, attention, limp, pregnancy, fatigue, tingling, intestinal causes, migraine, neurological alterations, sleep problems, female sex, traumatism and vision. B-IPQ, Brief-Illness Perception Questionnaire.

**Figure 2:**
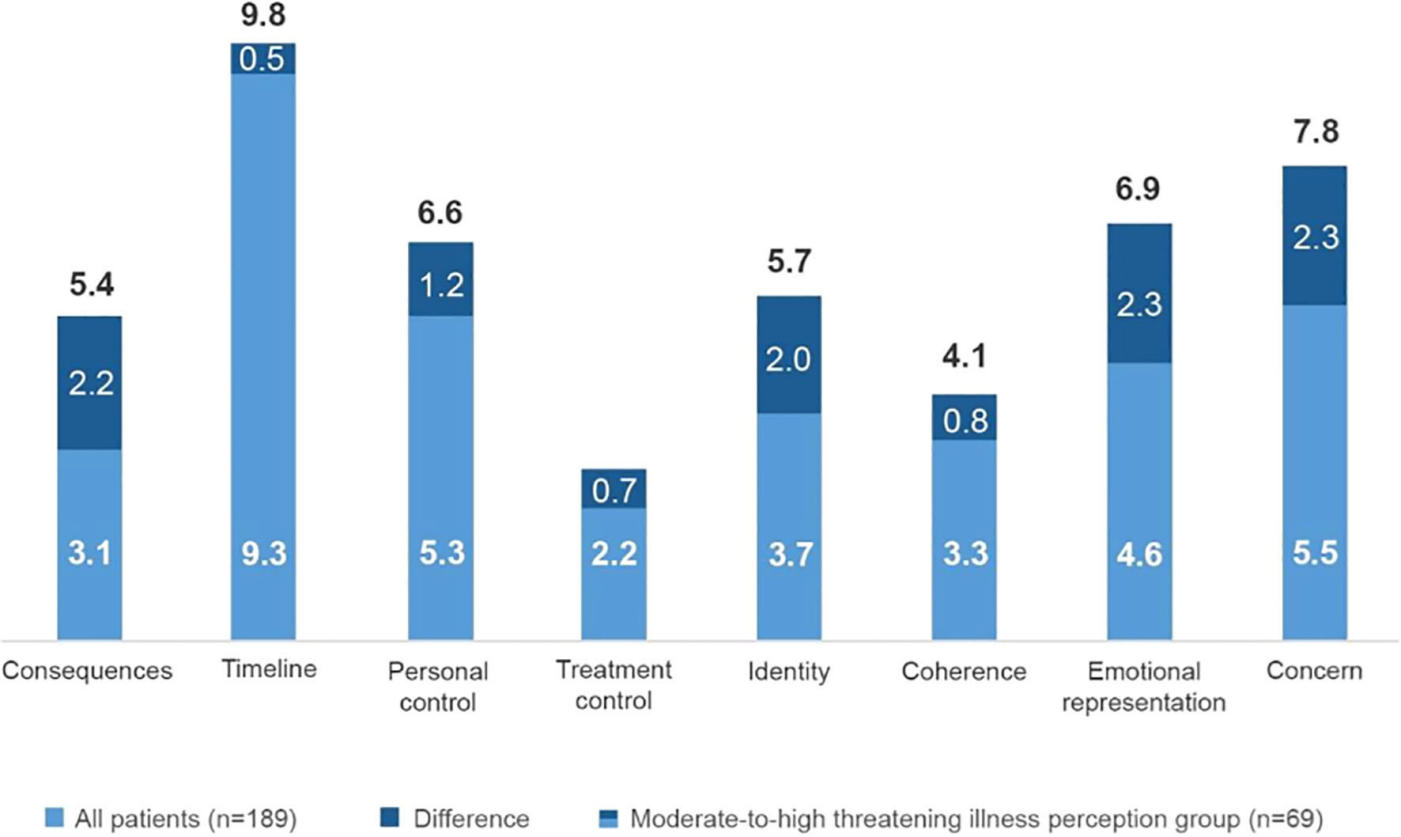
Patient perception of illness. Patients' illness perception in the different items of the Brief-Illness Perception Questionnaire.

A total of 36.5% of the patients (n=69/189) had a moderate-to-high threatening illness perception following the cut-off of the total score. When assessing the open question (item 9), a proportion of 47.6% (n=90/189) of patients thought that their disease was caused by psychological factors such as stress, anxiety, depression or nervousness, followed by unknown causes (17.5%), including chance or bad luck (**Figure 1**). The analysis of illness perception by its dimensions revealed that the most threatening aspect of multiple sclerosis for the patients was illness duration, followed by concerns from the disease, lack of personal control, and emotional impact. Nevertheless, the patients perceived that treatment helped to control their disease (**Figure 2**). Patients with a moderate-to-high threatening illness perception had poorer emotional representations (difference [Δ], Δ=2.3), concerns (Δ=2.3), consequences (Δ=2.2), and identity (Δ=2.0) beliefs when compared to the overall group, whereas coherence (Δ=0.8) and treatment control (Δ=0.7) perceptions did not differ much between groups (**Figure 2**).

The bivariate analyses suggested that patients were significantly more likely to have a threatening disease perception if they were unemployed, had received symptomatic treatment, had a higher EDSS score, poorer perception of their hand dexterity and gait, higher perceptions referred to pain, fatigue, symptom severity, hopelessness, and poorer perception of their quality of life. The same applied to patients reporting workplace barriers, cognitive complaints, and probable cases of anxiety, depression, and stigma.

Higher EDSS scores, greater symptom severity, a poorer psychological quality of life, perception of stigma, and greater hopelessness were identified as predictors of moderate-to-high threatening illness perception (**Table 3**).

**Table 3:** Predictors of moderate-to-high threatening illness perception.

Multivariate analysis - Variables	OR	95% CI	p-value
EDSS	2.44	1.28-5.13	0.011
SyMS	1.21	1.09-1.36	0.001
MSIS-29 psychological	1.06	1.02-1.09	0.003
SSCI-8 (= 8 vs > 8)	3.37	1.16-10.80	0.032
STHS total score trait	41.18	6.94-388.83	0

CI, Confidence interval; EDSS, Expanded Disability Status Scale; MSIS-29, Multiple Sclerosis Impact Scale; OR, Odds ratio; SSCI-8, Stigma Scale for Chronic Illness; STHS, State-Trait Hopelessness Scale; SyMS, SymptoMScreen.

## 4 Discussion

Identifying patient beliefs and expectations at the beginning of the disease might be of importance for promptly implementing educational strategies aimed at influencing patient adjustment. These perceptions can have an influence into patients’ coping style, adopting an active role to employ adaptative strategies that facilitate overcoming stress or on the contrary developing an avoidant/maladaptive style, as well as can lead to employing the right coping strategy of problem-solving and task-oriented, while avoiding the others in seek of greater quality of life and well-being over the long term (37). This study addresses a critical knowledge gap, as evidence on illness perceptions in early-stage RRMS patients with low levels of disability is scarce. We assessed patient illness perceptions in a population with a disease duration of less than three years from the first symptom and with very low disability. We found that more than a third of these patients experienced moderate-to-high threatening illness perception, with disability, symptom severity, and psychological factors being predictors of this condition.

A recent systematic review of previous works reported that a greater emotional impact, number of symptoms, higher perception of negative consequences of the disease for the life of the patient, and attributing the cause of the disease to psychological factors, were related to poorer outcomes in terms of well-being, adjustment, quality of life, or fatigue (18). In contrast, stronger perceptions of personal control over the illness, disease comprehension, and attributing the cause of the disease to external factors – relieving the sense of guilt – were seen to be related to better outcomes and possibly to more effective management of the disease and a lower level of distress (18). In our study, we found that having a moderate-to-high threatening illness perception was mainly driven by stronger perceptions in those dimensions related to poorer outcomes (illness duration, lack of personal control, and emotional impact), including concerns. In some of them, the range of improvement might be minimal, such as perceptions of illness duration, as it is a chronic condition. However, the others might benefit from tailored psychoeducational interventions that address emotional health, social support, and adaptive coping strategies (8, 37). Although a 63.5% of patients were not categorized as having a moderate-to-high threatening-illness disease, they might benefit as well from these assessments and interventions, as the highest scores were placed on the same dimensions, with a lower impact. Our identification of greater disability and symptom severity as predictors of threatening perception in an early and scantly disabled population is in line with the data from recent studies, where disease severity has been significantly associated with negative illness perceptions in multiple sclerosis patients with mild disability, playing a crucial role in terms of sexual dysfunction (38). Physicians should pay attention to these beliefs in the early disease stages, as negative beliefs regarding concerns, treatment and serious consequences of the disease might increase with a longer duration of illness (39). Furthermore, previous studies have found that illness perception mediates an association between a poorer perception of physical condition and negative treatment efficacy beliefs (40). Interestingly, while a higher proportion of patients with a threatening perception of their illness had poorer perceptions of their physical condition, we did not observe substantial differences in treatment control beliefs. This finding may suggest that early-stage patients maintain a sense of treatment efficacy, which could be leveraged to foster engagement in disease-modifying therapies and rehabilitation strategies (41). Moreover, poorer illness perceptions have been related to psychological factors (16, 42, 43). We found that psychological factors such as the perception of stigma, greater hopelessness, and poorer psychological quality of life were predictors of a threatening condition perception, and that almost half of the patients thought their disease had a psychological cause. These findings emphasize the importance of early psychological assessment to identify these limiting factors. Interventions like meditation, mindfulness or yoga can be implemented to reduce those that are more manageable such as anxiety, nervousness, and stress (44). They also point to the potential benefits of establishing education programs to address misattributions about the causes of the disease. Both measures may help reduce stigma and improve illness perception, enhancing adjustment to the disease and ultimately having a positive impact on emotional well-being. Besides psychological causes, one out of four patients in our study thought that their MS was caused by random events or bad luck. These misattributions of causes of MS reflect misinformation that may come from internet searches, as it is one of the most common sources of information used by patients to find MS causes or risk factors, with the difficulty of recognizing reliable information (45, 46). Healthcare professionals might face the challenge of transforming these untrue claims and beliefs to scientific-based information through guidance of patients and caregivers toward prudent searches, by avoiding advertisement and personal experiences that may not apply to others, while also looking for easily understandable, referenced content (47).

Considering these findings, the implementation of educational programs, adapting coping strategies, and establishing psychological intervention at the time of diagnosis, may be crucial for a comprehensive understanding of the disease and its evolution - including the recognition and management of symptoms, awareness of treatment options, and the impact of lifestyle factors (48, 49) - placing emphasis on those individuals with greater disability, symptoms, and a poorer psychological status, as evidenced in our study. Consequently, patients will have more tools to better face their disease, enhancing their illness perception, achieving psychological protection, and increasing their self-management to actively participate in the shared decision-making process throughout their disease (11, 16).

Our study has some limitations. Firstly, its cross-sectional design did not allow us to assess changes in patient illness perception over time and after implementing interventions, since the study involved a single visit. Secondly, external factors such as caregivers’ perception were not assessed in this study and might be contributors to the outcome (42). However, the study provides relevant information on early-stage RRMS patient illness perception in a key moment, namely the period surrounding the diagnosis, and on patient adjustment to the new disease.

## 5 Conclusions

Threatening illness perceptions are common among patients with early RRMS and may be related to greater disability and symptoms, a poorer psychological quality of life, the perception of stigma, and hopelessness. Identifying these beliefs and their associated factors after the diagnosis, and establishing individualized interventions, may help patients deal with their condition over the long term.

## Data Availability

The raw data supporting the conclusions of this article will be made available by the authors, without undue reservation.
